# Advances in diffuse glioma assessment: preoperative and postoperative applications of chemical exchange saturation transfer

**DOI:** 10.3389/fnins.2024.1424316

**Published:** 2024-07-31

**Authors:** Hua-Zhen Deng, Han-Wen Zhang, Biao Huang, Jin-Huan Deng, Si-Ping Luo, Wei-Hua Li, Yi Lei, Xiao-Lei Liu, Fan Lin

**Affiliations:** ^1^Shantou University Medical College, Shantou City, China; ^2^Department of Radiology, The First Affiliated Hospital of Shenzhen University, Health Science Center, Shenzhen Second People’s Hospital, Shenzhen, China; ^3^Department of Radiology, Guangdong Provincial People’s Hospital (Guangdong Academy of Medical Sciences), Southern Medical University, Guangzhou, Guangdong, China; ^4^Department of Radiology, Shenzhen Samii Medical Center (The Fourth People’s Hospital of Shenzhen), Shenzhen, China

**Keywords:** amide proton transfer imaging, adult-type diffuse glioma, preoperative diagnosis, postoperative assessment, precision medicine

## Abstract

Chemical Exchange Saturation Transfer (CEST) is a technique that uses specific off-resonance saturation pulses to pre-saturate targeted substances. This process influences the signal intensity of free water, thereby indirectly providing information about the pre-saturated substance. Among the clinical applications of CEST, Amide Proton Transfer (APT) is currently the most well-established. APT can be utilized for the preoperative grading of gliomas. Tumors with higher APTw signals generally indicate a higher likelihood of malignancy. In predicting preoperative molecular typing, APTw values are typically lower in tumors with favorable molecular phenotypes, such as isocitrate dehydrogenase (IDH) mutations, compared to IDH wild-type tumors. For differential diagnosis, the average APTw values of meningiomas are significantly lower than those of high-grade gliomas. Various APTw measurement indices assist in distinguishing central nervous system lesions with similar imaging features, such as progressive multifocal leukoencephalopathy, central nervous system lymphoma, solitary brain metastases, and glioblastoma. Regarding prognosis, APT effectively differentiates between tumor recurrence and treatment effects, and also possesses predictive capabilities for overall survival (OS) and progression-free survival (PFS).

## 1 Introduction

Chemical Exchange Saturation Transfer (CEST), initially known as chemical exchange saturation transfer, was first introduced in Ward and Balaban (2000). They successfully generated the first contrast images using this technique. It is a novel magnetic resonance imaging (MRI) technique developed based on the principles of magnetization transfer technology and chemical exchange theory. The imaging mechanism employs specific off-resonance saturation pulses to comprehensively presaturate target molecules, facilitating the detection of their chemical exchange properties. This saturation, facilitated by chemical exchange, subsequently alters the signal intensity of the surrounding free water molecules. By detecting these water signals, information about the targeted substances can thus be indirectly inferred (van Zijl and Yadav, 2011). Currently, well-established techniques include chemical exchange saturation transfer (CEST) imaging for glutamate (Glu) (Schuenke et al., 2017), amide proton transfer (APT) (De Cocker et al., 2018), and glycosaminoglycan (GAG) (Krishnamoorthy et al., 2017). These techniques have shown significant progress in areas such as epilepsy, gliomas, and articular cartilage injury.

The APT imaging is a specialized type of CEST that utilizes specific frequency presaturation pulses to selectively excite amide protons on protein polypeptide chains. In APT imaging, the saturation state of amide protons is transferred to free water protons. When this process is repeated multiple times, it leads to a significant reduction in the signal of free water protons. The magnitude of the APT signal is represented by the attenuation of the water signal observed before and after the application of the saturation pulse. The degree of water signal attenuation is determined by the exchange rate between the two proton types, which is contingent upon the concentration of amide protons and the pH of their surrounding environment. Within a stable internal environment, an increase in protein content leads to a higher exchange rate between protons, resulting in a more pronounced APT signal. Consequently, APT imaging demonstrates sensitivity to changes in protein concentration (Zhou et al., 2019, 2022; Jiang et al., 2023b).

Gliomas, the most prevalent primary brain tumors, account for around 30% of primary central nervous system neoplasms (Ostrom et al., 2022). The crucial anatomical location, aggressive growth patterns, and high malignant potential render gliomas particularly deleterious to human health (Raab et al., 2022). Accurate diagnosis is pivotal for guiding clinical treatment strategies. In the 2021 fifth edition of the World Health Organization (WHO) classification of central nervous system tumors, gliomas are categorized into adult and pediatric types for the first time. They are further subcategorized into diffuse and localized based on their distinct growth patterns and infiltration characteristics. High-grade gliomas (HGG) may not display notable contrast enhancement, and the enhancement patterns do not always align with the degree of malignancy. Substantial variations in protein expression levels exist among tumor cells across various grades of gliomas. Consequently, APT imaging facilitates the detection of tumor protein expression, enables the assessment of tumor grading, and discriminates between treatment-induced damage and tumor recurrence (Louis et al., 2021). This paper reviews the application of APT imaging in the preoperative diagnosis and postoperative assessment of gliomas over the past 5 years.

## 2 The glioma grading

According to the 2021 WHO criteria, gliomas are divided into localized and diffuse types, of which diffuse gliomas are divided into adult and pediatric types. According to different molecular subtypes, grades 1 and 2 were classified as low grade, and grades 3 and 4 were classified as high grade. The histological grading currently employed in clinical practice is based on the immunohistochemical grading scheme from the 2007 edition. In contrast, the 2016 histological classification refers to the Central Nervous System (CNS) IV (Table 1). The role of APT imaging in glioma grading has garnered considerable attention through extensive research endeavors. Notably, Sotirios et al.’s, 2020 meta-analysis accentuated the criticality of accurately grading gliomas’ malignancy. Studies conducted by Suh et al. (2019), Kang et al. (2020), and Hou et al. (2023a) have consistently revealed that APT signals are significantly elevated in HGG as compared to their low-grade counterparts. This finding underscores the high sensitivity and specificity of APTw MRI in discriminating between these grades. Further, in a study on APTw-guided stereotactic biopsy, Jiang et al. (2017) found that the APTw signal intensity at the biopsy sites for each patient, as well as the maximum APTw value across all biopsy sites, were significantly higher in high-grade specimens compared to low-grade ones. Similarly, Durmo et al. (2020) demonstrated that the combination of the average, maximum, and range of APTw signals could distinguish between low-grade gliomas (LGG) and HGG, with a corresponding AUC of 0.958. These studies indicate the effectiveness of different APTw signal metrics in the diagnostic grading of gliomas. Contrarily, in non-enhancing gliomas, Togao et al.’s (2017) study demonstrated that APT achieved moderate diagnostic performance in glioma grading (AUC = 0.811). Schön et al. (2020) discovered that APTw hot-spot volumes (HSV) was most elevated in glioblastomas.

**Table 1:** 2007 and 2016 WHO CNS classification systems.

Grade	2007 type	Immunohistochemical markers	2016 type	Immunohistochemical markers
I	Pilocytic astrocytoma	GFAP+	Pilocytic astrocytoma	GFAP+
II	Diffuse astrocytoma	GFAP+, p53+/−	Diffuse astrocytoma, IDH mutant	GFAP+, IDH1/2+, p53
II	Oligodendroglioma	Olig2+, IDH1 R132H+	Oligodendroglioma, IDH mutant and1p/19q co-deletion	Olig2+, IDH1/2+,1p/19q−
II	Oligoastrocytoma	GFAP+, Olig2+	Oligoastrocytoma	GFAP+, Olig2+
III	Anaplastic Astrocytoma	GFAP+, Ki-67+, p53+	Anaplastic astrocytoma, IDH mutant	GFAP+, Ki-67+, IDH1/2+, p53+
III	Anaplastic oligodendroglioma	Olig2+, Ki-67+, IDH1 R132H+	Anaplastic oligodendroglioma, IDH mutant and1p/19q co-deletion	Olig2+, Ki-67+, IDH1/2+,1p/19q−
III	Anaplastic oligoastrocytoma	GFAP+, Olig2+, Ki-67+	Anaplastic oligoastrocytoma	GFAP+, Olig2+, Ki-67
IV	Glioblastoma	GFAP+, EGFR+, Ki-67+, p53+/−	Glioblastoma, IDH wild-type	GFAP+, EGFR+, Ki-67+ p53+/−, IDH1/2-
IV	Giant cell glioblastoma	GFAP+, p53+, Ki-67+	Giant cell glioblastoma	GFAP+, p53+, Ki-67+
IV	Gliosarcoma	GFAP+, BRAF V600E+	Gliosarcoma	GFAP+, BRAF V600E+

Compared to other imaging modalities, APTw MRI demonstrates distinctive advantages in the grading of gliomas. As concluded by Togao et al. (2017) and Xu et al. (2021), APT values provide more substantial insights than alternative imaging metrics for this specific purpose. Conversely, Choi et al. (2017) emphasized the benefits of combining APT with PWI and DWI techniques. In their respective studies, Bai et al. (2017) and Su et al. (2021) utilized distinct APTw signal metrics and discovered that the diagnostic models for discriminating between LGG and HGG both exhibited an AUC exceeding 0.75. Choi et al. (2017) found that the combined use of APT signal and ADC significantly improved diagnostic accuracy compared to using ADC alone (AUC = 0.910), while the combination of APT signal and rCBV did not enhance differentiation ability. Similarly, Zou et al. (2018) discovered that the combined use of APTw and IVIM exhibited the best diagnostic performance (AUC = 0.986), suggesting that APTw and IVIM, as two promising complementary sequences to conventional MRI, might be highly valuable in distinguishing LGG from HGG (Choi et al., 2017).

Warnert et al. (2022) emphasize the efficacy of APT imaging in glioma assessment, citing its ability to distinctly delineate glioma progression due to its high sensitivity. Complementing this, a collaborative study between Huazhong University of Science and Technology and the University of Illinois has proposed an advanced APT imaging technique based on Z-spectrum fitting, offering enhanced accuracy in glioma grading (Warnert et al., 2022). In a detailed analysis, Liu et al. (2023) evaluated the performance of magnetization transfer ratio asymmetry (MTRasym) across various frequency offsets, employing three Lorentzian functions to represent direct water saturation (DS), APT, and the combined effects of semisolid magnetization transfer and nuclear Overhauser enhancement (MT and NOE). Their findings indicated that the AUC for fit-based measurements was significantly higher compared to non-fit approaches (Liu et al., 2023). In another vein, Wada et al. (2023) analyzed CEST imaging data from 28 patients. Their investigation, encompassing different dimensional imaging techniques, revealed that HGGs consistently showed higher MTRasym values than LGGs, irrespective of the imaging method used. Furthering these insights, Paech et al. (2018) research, which utilized CEST MRI on a 7T whole-body scanner, demonstrated the reliability of the dns-APT index in differentiating LGG from HGG. The study identified an average dns-APT value of 1.88 as the optimal threshold for classification, resulting in a sensitivity of 71% and a specificity of 100% (Paech et al., 2018).

Recent advancements indicate that integrating APTw imaging with radiomics technology enhances glioma grading accuracy. Sartoretti et al.’s (2021) study demonstrated this through radiomics feature extraction and analysis of APTw MR Imaging. Wu et al. (2023) also verified the high precision of the CESTR-radiomics model in classifying gliomas. These findings suggest significant advantages of APT imaging in glioma grading, particularly in non-enhancing gliomas. Combining advanced imaging techniques with image omics analysis can yield more accurate glioma diagnoses, contributing valuable insights to precision medicine (Table 2).

**Table 2:** Amide proton transfer imaging used in the classification of the malignant degree of glioma.

Reference	Method of application	Sample size	AUC	Conclusion
Choi et al. (2017)	APT, DTI, MRI	46	0.867	APT imaging may be a useful imaging biomarker that adds value to the ADC for discriminating between low- and high-grade gliomas.
Jiang et al. (2017)	APT	24	0.766	APTw imaging detects high-cellularity areas and aids in identifying high-grade regions in heterogeneous gliomas, enhancing neurosurgical accuracy
Bai et al. (2017)	APT, ADC, CBF	44	0.825	Amide proton transfer magnetic resonance imaging is a promising method for predicting the grading and cellularity of gliomas.
Zou et al. (2018)	APT, IVIM	51	1.00	APTW and IVIM, as two promising supplementary sequences for routine MRI, could be valuable in differentiating LGGs from HGGs.
Paech et al. (2018)	7 T CEST	31	0.83	Relaxation-compensated multi-pool CEST MRI, including dns-APT, distinguishes LGG from HGG non-invasively
Suh et al. (2019)	APT	353	NA	APTw MRI excels in differentiating low- and high-grade gliomas, offering reliability in clinical grading
Durmo et al. (2020)	APT	22	0.93	Incorporating APTw-images in pre-operative evaluation improves differentiation of LGGs from HGGs, enhancing patient management and treatment
Kang et al. (2020)	APT, ASL, DWI	27	0.84	APT and DWI differentiate HGGs from LGGs; APT, DWI, and ASL combination enhances discrimination.
Schön et al. (2020)	APT, PET, DSC	46	NA	APTw was lower in lower-grade gliomas compared with glioblastomas. APTw meaningfully contributes to biological imaging of gliomas.
Sartoretti et al. (2021)	APT	48	0.868	Radiomics feasible for APTw imaging; high accuracy in distinguishing primary glial brain tumors from metastases
Su et al. (2021)	APT, MTC, MRSI	81	0.854	Glioma patients exhibit strong inter-metabolite correlations, linked to grade and proliferation; multi-IMCCs model enhances diagnosis
Xu et al. (2021)	APT.DKI	51	0.986	APT imaging excels over DKI for glioma grading and IDH mutation prediction, enhancing diagnosis and treatment
Warnert et al. (2022)	APT	18	NA	Study highlights need for whole-tumor coverage to explore APT signal in non-enhancing glioma heterogeneity
Guo H. et al. (2022)	APT, DTI, DKI, MAP, NODDI	62	0.924	APT surpasses DTI, DKI, MAP, NODDI in glioma IDH genotype diagnosis; APT5 identifies grading and genotyping effectively
Hou et al. (2023a,b)	3D-APTW, 3D-Pcasl	81	0.90	Combining 3D-APTW with 3D-pCASL imaging aids in assessing glioma grading
Liu et al. (2023)	APT	38	0.90	Fit-free CEST metrics at 3 ppm outperform others in grading brain gliomas, with fit-based metrics offering improved differentiation.
Liu et al. (2023)	APT	28	0.78	3D CEST imaging at 3.5 ppm provides MTRasym per slice, with diagnostic performance like 2D CEST
Wu et al. (2023)	APT	129	0.88	APTw imaging and radiomics hold promise in predicting IDH mutation and grading adult diffuse glioma

## 3 Diagnosis of molecular subtypes of gliomas

**Figure 1:**
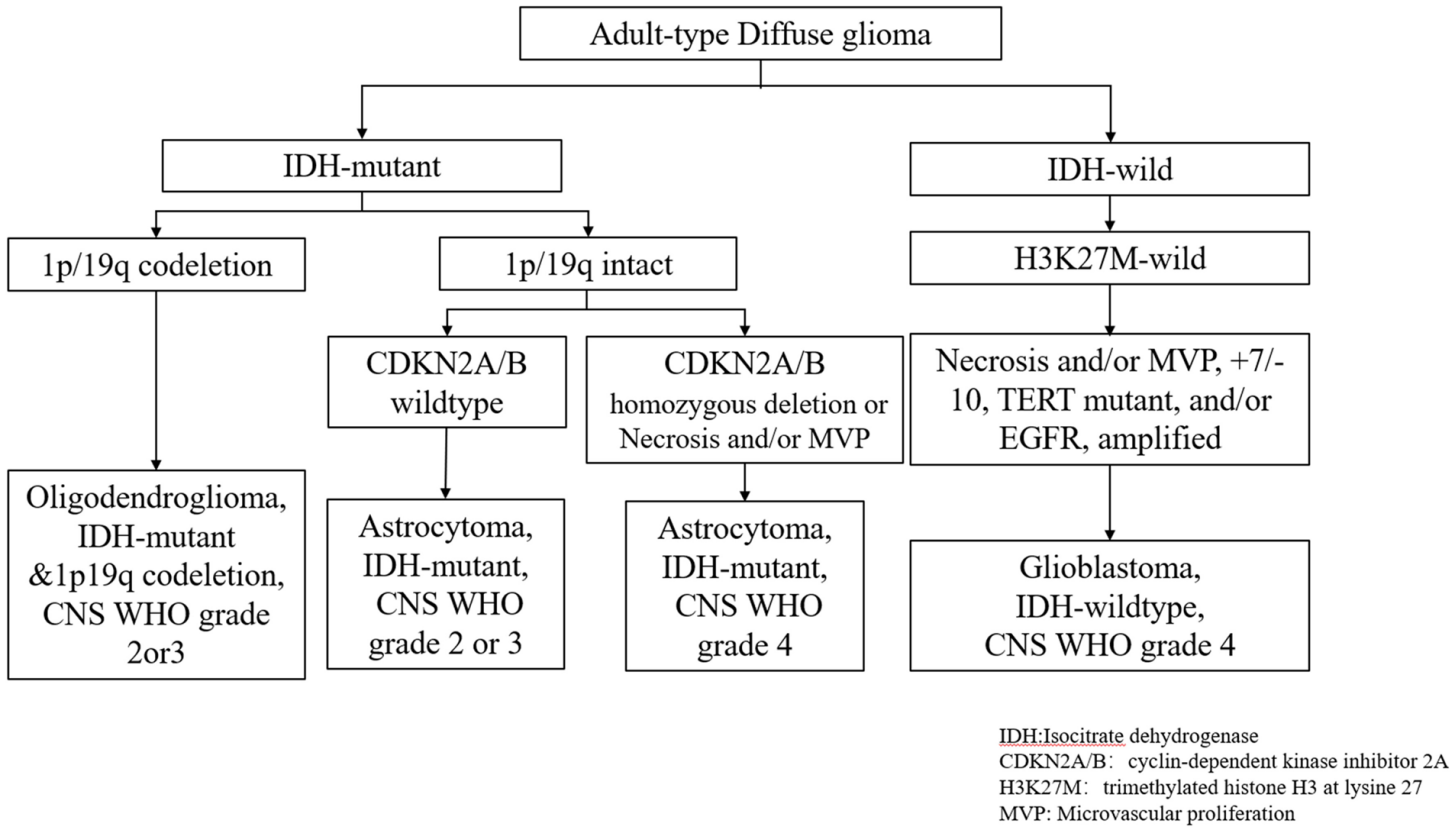
Molecular diagnostic route of adult diffuse glioma.

In 2021, the WHO CNS5 was released, which categorized tumors into more biologically and molecularly defined pathological types, placing additional emphasis on molecular expression or genetic alterations. These updated classifications will allow clinicians to better understand the prognosis and optimal treatment of patients with specific CNS tumors, and it will also facilitate the participation of more homogeneous patient populations in clinical trials, facilitating the evaluation of new therapies. Adult diffuse gliomas can be divided into three categories according to the presence or absence of IDH mutation and 1p and 19q (1p/19q) deletion: astrocytoma, IDH-mutant; Oligodendroglioma, IDH mutation with 1P19q co-deletion; Glioblastoma, IDH wild-type (Wagner et al., 2024) (Figure 1).

### 3.1 Identification of IDH mutation status

The IDH mutation status is crucial for prognostication in glioma patients. Mutations in IDH genes lead to reduced enzyme activity, converting alpha-ketoglutaric acid (alpha-KG) to 2-hydroxyglutaric acid (2-HG). This alteration not only inhibits α-KG enzyme activity, affecting glutamate and glucose metabolism but also suppresses tumor cell proliferation (Louis et al., 2016). Conversely, gliomas with IDH wild-type status are more invasive and linked to poorer prognoses. In WHO CNS5, glioma patients with IDH wild-type will be directly classified as glioblastoma. Recent research has leveraged APTw imaging to differentiate IDH statuses. For instance, Joo et al. (2019) observed that IDH wild-type grade 3/4 gliomas exhibited significantly higher APTw intensities than IDH mutant gliomas in a study involving 71 HGG patients at 3T MRI. Paech et al. (2018) using 7T CEST MRI imaging indicated that dns-APT CEST effectively predicts IDH mutation status. Guo H. et al. (2022) focused on various imaging techniques for glioma grading and IDH state diagnosis, finding that multiple imaging parameters were notably higher in IDH wild-type gliomas. Su et al. (2022) introduced a model utilizing various Z-spectrum contrasts, which exhibited superior performance in identifying IDH MUT and WT states. Moreover, Yuan et al. (2022) research exploring a convolutional neural network (CNN) combined with ultra-high field 7T CEST imaging made significant strides in the preoperative identification of IDH status. Similarly, Wu et al. (2023) study confirmed the effectiveness and robustness of an APTw imaging-based machine learning model in predicting IDH mutation status via MRI.

### 3.2 Identification of MGMT methylation mutation status

The MGMT promoter methylation is a pivotal prognostic marker in glioblastoma, influencing patient responsiveness to alkylating chemotherapy agents like temozolomide (TMZ). Methylation of this gene’s promoter leads to reduced O6 -methylguanine-DNA methyltransferase (MGMT) protein expression, enhancing chemotherapy sensitivity and improving prognosis (Louis et al., 2016). Su et al. (2018), with a sample of 42 subjects, examined the relationship between MGMT immunostaining results and APTw characteristics. They identified significant differences in APTw features between MGMT-positive and -negative tumors, with an impressive predictive value (97.3%) and an AUC of 0.849 (Su et al., 2018). Jiang et al. (2018) investigated the use of CEST-MRI, including APTw, for identifying MGMT promoter methylation status in glioblastoma. They found that unmethylated glioblastomas exhibited higher CEST signals (1–4.5 ppm range) with the 90th percentile APTw value yielding the highest AUC (0.856) and an average APTw value accuracy of 83.3% in predicting methylation status (Jiang et al., 2018). However, contrasting findings were presented by Paech et al. (2018), who utilized 7T CEST MRI technology and observed no significant difference (*p* > 0.05) in CEST signals (including DNS-APT) between different MGMT promoter methylation statuses. A similar conclusion was drawn in Joo et al.’s (2019) study. These discrepancies indicate that while CEST/APT imaging shows potential in distinguishing MGMT methylation status in glioblastoma, the results are not yet definitive. Future studies are needed to validate the effectiveness of these imaging techniques and to understand their underlying mechanisms better.

### 3.3 Identification of 1p/19q co-deletion states

The complete co-deletion of chromosome 1p/19q is a critical molecular genetic marker in diagnosing oligodendroglioma. This genetic feature is notably associated with a better response to chemoradiotherapy and improved prognosis compared to non-co-deletion gliomas (Engelhard et al., 2003). Addressing this issue, Yao et al. (2020) conducted a 3T CEST imaging study involving 76 glioma patients, focusing on the extracellular acidity in WHO grade II and III gliomas with 1p/19q co-deletion. Their findings revealed a significantly lower MTRasym value in gliomas with co-deletion compared to those without, with an AUC of 0.85. This suggests that MTRasym biomarkers could serve as a non-invasive method for identifying this co-deletion (Yao et al., 2020). Moreover, Su et al. (2022) investigated Z-spectrum MRI methodologies, specifically evaluating the disparities between direct saturation of water (DSW) and CEST@2 ppm Z-spectrum contrasts within IDH-mutant gliomas. Their research revealed that gliomas characterized by 1p/19q co-deletion demonstrated reduced signal intensity (Su et al., 2022). These findings are significant for distinguishing tumor subtypes, thus playing a crucial role in clinical diagnosis and treatment decision-making (Table 3).

**Table 3:** Amide proton transfer imaging used in the preoperative determination of the molecular type of glioma.

Reference	Method of application	Molecular type	Sample size	AUC	Conclusion
Paech et al. (2018)	7T CEST	IDH and MGMT	31	0.97	Relaxation-compensated CEST MRI, especially dns-APT, predicts IDH status and differentiates LGG from HGG non-invasively
Jiang et al. (2018)	APT	MGMT	18	0.825	APTw signal metrics show potential as imaging biomarkers for identifying MGMT methylation status in GBM
Su et al. (2018)	APT, IHC	MGMT	42	0.849	APTw characteristics may be promising non-invasive preoperative markers for predicting IHC MGMT expression in gliomas
Joo et al. (2019)	APT	IDH	71	NA	High APT signal predicts poor HGG prognosis, offering incremental value over clinical factors and IDH mutation status
Yao et al. (2020)	APT	1p19q	76	0.85	Amine CEST-MRI, indicating less acidic 1p/19q co-deleted gliomas, could be a non-invasive biomarker for co-deletion status
Xu et al. (2021)	APT.DKI	IDH	51	0.92	APT imaging surpasses DKI in glioma grading and IDH mutation prediction, aiding in precise diagnoses and treatments
Guo H. et al. (2022)	APT, DTI, DKI, MAP, and NODDI	IDH	62	0.870	APT outperforms DTI, DKI, MAP, and NODDI in glioma IDH genotyping, making APT5 a promising classification biomarker
Yuan et al. (2022)	CEST, MRS	IDH	18	0.95	7T CEST/MRS combination, as a non-radioactive method, could redefine guidance for glioma resection and irradiation
Su et al. (2022)	Multi-parametric Z-spectral MRI	IDH and 1p19q	113	0.903/0.836	Multi-parametric Z-spectral MRI is a comprehensive, noninvasive technique for clinical glioma stratification
Yuan et al. (2023)	7T CEST	IDH	84	0.887	7T CEST and structural MRI combined enhance preoperative IDH mutation diagnosis, showcasing ultra-high-field CEST and CNN’s clinical potential
Wu et al. (2023)	APT	IDH	129	0.87	APTw imaging and radiomics offer a promising quantitative method for predicting IDH mutation and grading diffuse glioma

## 4 Prognosis of high-grade gliomas

### 4.1 Differentiating postoperative progression from post-treatment changes

In glioma treatment, imaging is pivotal for monitoring tumor progression (TP) or treatment-related changes (TRC). However, differentiating TRC from TP in treated glioma patients is challenging due to their similar clinical symptoms and morphological imaging features. In addressing this concern, Meissner et al. (2019) conducted a study to explore the capability of CEST MRI in the early evaluation of chemoradiotherapy response among glioma patients. Their research, involving 12 patients treated with 7T CEST MRI, demonstrated that the rNOE signal could markedly differentiate between stable and progressive disease following treatment. This finding highlights the potential of CEST MRI in the early assessment of treatment efficacy (Meissner et al., 2019). Park et al. (2020) evaluated the potential of alterations in APT signal intensity following antiangiogenic therapy to predict early treatment response in recurrent glioblastoma. Their findings indicated that decreases in APT signal intensity within 4–6 weeks post-treatment were associated with extended progression-free survival at 12 months (Park et al., 2020).

Further, Guo P. et al. (2022) integrated APTw MRI data into structural MR images and developed a new CNN model to differentiate between tumor progression and response. The results showed that the AUC for slice-level classification increased from 0.88 to 0.90, and for scan-level classification, the AUC increased from 0.85 to 0.90, significantly enhancing the classification capability (Guo P. et al., 2022). Guo P. et al. (2022) compared the diagnostic performance of DWI, ASL, MRI, and APTw imaging in distinguishing between recurrent tumor (TuR) and treatment effect (TrE). They found that the APTw effect and relative cerebral blood flow (rCBF) were significantly higher in recurrent tumors compared to treatment effect lesions. ASL and APT imaging performed better in differentiating TuR from TrE, although ASL imaging had a lower signal-to-noise ratio, longer imaging times, and lower sensitivity to areas with slower blood flow (Guo P. et al., 2022). Park et al. (2021) evaluated the incremental value of APTw over DTI, DSC, and DCE in distinguishing recurrent glioma from treatment-induced changes. The results showed that higher APT signals were associated with recurrent gliomas. Adding APT signals significantly improved the diagnostic performance of models based on ADC, FA, and nCBV (Park et al., 2021). Chen et al. (2022) conducted a Bayesian bivariate meta-analysis to evaluate the standalone and additional value of APT imaging. The results indicated that combining APTw imaging parameters further improved diagnostic performance, with pooled sensitivity and specificity of 0.91 and 0.92, respectively (Chen et al., 2022). Huang et al. (2023) utilized DWI, SWI, pcASL, and APTw imaging in their study and found that DWI was sensitive to motion artifacts and magnetic field inhomogeneity, resulting in lower specificity. Several histogram parameters from APTw and pcASL imaging could significantly distinguish between treatment effects and tumor recurrence. The regression model combining all significant histogram parameters showed the best performance, with an AUC of 0.89 (Huang et al., 2023). Hou et al. (2023b) prospectively evaluated the diagnostic performance of 3D-APTw, 3D-PcASL, and DWI in distinguishing true progression from treatment response in patients with post-treatment malignant gliomas. The results indicated that 3D-APTw exhibited good diagnostic performance with an AUC of 0.911. Moreover, combining rAPTw values and rCBF values achieved even better diagnostic performance, with an AUC of 0.951 (Hou et al., 2023b). Jiang et al.’s (2019) study underscored APT imaging’s significance in tumor diagnosis and treatment evaluation. Analyzing 64 case-section specimens from 21 glioma patients, they found that an APTw intensity cut-off value of 1.79% had 94.4% sensitivity and 100% positive predictive value for identifying glioma recurrence (Jiang et al., 2019). Additionally, incorporating 525 radiomics features from APTw and structural MR Images of 86 patients into their model, Jiang et al. (2023a) achieved an 85.0% sensitivity and 100% specificity in distinguishing tumor recurrence from treatment response. Paprottka et al. (2021) combined [18F]-FET-PET and MRI imaging with DSC perfusion (including APTw) and deep learning, finding this approach to be a promising tool for objective response assessment in glioma. Collectively, these studies highlight the significant potential of APTw imaging and other advanced MRI techniques in evaluating treatment response and identifying tumor progression in gliomas, providing crucial insights for personalized glioma treatment. Despite these studies demonstrating the potential of APTw imaging and multiparametric MRI, limitations remain, such as small sample sizes, variations in imaging parameters and analysis methods, and issues related to the computational demands and interpretability of deep learning models.

Future research should focus on standardizing imaging and analysis methods and validating the efficacy of these techniques in larger, multicenter studies. Additionally, optimizing the training and interpretability of deep learning models is crucial. Combining various imaging modalities, such as APTw, ASL, DWI, and PWI, can provide more comprehensive diagnostic information and improve diagnostic accuracy.

### 4.2 Prediction of OS or PFS

In the treatment and management of glioma, OS and PF are important indicators to measure the clinical efficacy. For HGG patients, due to its high malignant degree, OS and PFS accurate prediction is particularly important. Recent studies have begun to explore the use of CEST MRI techniques in this field. Paech et al. (2019) studied the 26 patients with HGG, evaluated the CEST MRI indicators [including the APT, relay nuclear Overhauser effect (rNOE)/NOE, end rNOE suppression APT (DNS – APT)] associated with OS/PFS. The results showed that APTw signal intensity was significantly correlated with OS (HR = 3.15, *p* = 0.02) and PFS (HR = 1.83, *p* = 0.009), while dns-APT index had the strongest correlation with PFS (HR = 2.61, *p* = 0.002) (Paech et al., 2019). The study by Joo et al. (2019) also found that high APT signal was a significant predictor of poor OS and PFS. von Knebel Doeberitz et al. (2023) evaluated the potential predictive value of APT and semi-solid magnetization transfer (ssMT) imaging for OS in HGG patients at the first follow-up after RT and found that APTw imaging had the strongest correlation with OS. MTconst and contrast enhanced residual glioma OS related participants of the organization (von Knebel Doeberitz et al., 2023). In addition, the study by Kroh et al. (2023) compared the potential of indicators based on different CEST techniques in predicting PFS in HGG patients and found that MTconst, PeakAreaAPT, and APTwasym were all associated with PFS. These findings suggest that CEST MRI technology and its various indexes in evaluating the survival of patients with HGG progression-free survival and has significant potential (Table 4).

**Table 4:** Amide proton transfer imaging used in differentiating postoperative progression from post-treatment changes and predicting OS or PFS.

Reference	Method of application	Sample size	AUC	Conclusion
Jiang et al. (2019)	APT	21	0.881	APTw imaging hyperintensity effectively identifies active malignant glioma with high sensitivity and specificity
Meissner et al. (2019)	APT	12	NA	CEST MRI may enable early response assessment in glioma patients.
Paech et al. (2019)	APT	26	NA	Relaxation-compensated APT MRI signal intensity is associated with overall survival and progression-free survival in newly diagnosed
Joo et al. (2019)	APT	71	0.766	High APT signal was a significant predictor of poor prognosis in HGG.
Liu et al. (2020)	APT, MRS, DWI, and ASL	30	0.93	Compared with DWI and MRS, ASL, and APT imaging techniques showed better diagnostic capability in distinguishing TuR from TrE.
Park et al. (2020)	APT MRI, DWI, and DSC	54	NA	Early decrease in APT signal intensity predicts better 12-month response and survival in recurrent glioblastoma.
Park et al. (2021)	APT, DTI, DSC, and DCE	36	0.92	APT imaging may be a useful imaging biomarker that adds value to DTI, DCE, and DSC parameters for distinguishing between recurrent gliomas and treatment-induced changes.
Paprottka et al. (2021)	[18F]-FET-PET, APTw, DSC	66	0.85	Joint analysis of [18F]-FET-PET, APTw, and DSC perfusion is promising for objective glioma response assessment
Guo H. et al. (2022) and Guo P. et al. (2022)	APT	98	0.90	Adding APTw MRI to structural MRI enhances CNN-based recurrent glioma classification at multiple levels
Chen et al. (2022)	APT	6	NA	APT imaging, combined with advanced MRI techniques, improves accuracy in differentiating TR from TE in post-treatment gliomas
Jiang et al. (2023a,b)	APT	86	0.895	Adding APTw-based radiomic features increased MRI accuracy in the assessment of the treatment response in post-treatment malignant gliomas.
Huang et al. (2023)	APT, DWI, SWI, pcASL	28	0.89	APTw images added value to other advanced MR images for the differentiation of treatment effect and tumor recurrence.
Hou et al. (2023a,b)	3D-APTW and 3D-Pcasl	48	0.951	3D-APTW imaging outperforms 3D-PcASL in distinguishing TP from TR, with combined rAPTW and rCBF values enhancing diagnosis
Kroh et al. (2023)	APT	72	0.79	MTconst enables differentiation of radiation-induced pseudoprogression from disease progression.
von Knebel Doeberitz et al. (2023)	APT	49	NA	APT and ssMT imaging post-radiotherapy at 3T correlates with overall survival in glioma patients
Kroh et al. (2023)	APT	72	0.79	MTconst, PeakAreaAPT, and APTwasym imaging predict clinical outcome by means of progression-free survival.

## 5 Differential diagnosis

### 5.1 Differentiating HGG from meningioma

Accurate differentiation between HGG and meningioma is crucial in the diagnosis of nervous system disorders. Both conditions often exhibit moderate to pronounced enhancement in traditional imaging, making it challenging to distinguish them based solely on the degree of enhancement. Addressing this challenge, Zhang et al. (2022) embarked on a study to identify more effective differentiation techniques. Their research highlighted the utility of APTmean, the APT ratio relative to enhanced images (RAPT/E), and the APT ratio relative to T2-weighted imaging (RAPT/T2) in differentiating HGG from meningiomas. The study revealed that APTmean values in HGG were significantly higher than in meningiomas, with HGG demonstrating a more pronounced range on APT images. These findings indicate that APTmean is a potent tool in distinguishing between HGG and meningiomas, offering a significant advantage over traditional imaging methods (Zhang et al., 2022).

### 5.2 Differentiating HGG from primary central nervous system lymphoma (PCNSL)

Distinguishing primary central nervous system lymphoma (PCNSL) from HGG poses a significant challenge in neuroimaging. Both conditions often exhibit similar Gadolinium (Gd)-enhanced masses and peripheral edema in conventional MR images (Tang et al., 2011). To enhance diagnostic accuracy, researchers are exploring advanced imaging techniques. Jiang et al. (2016) analyzed APT imaging in 11 lymphoma patients and 21 HGG patients, correlating findings with postoperative pathological results. They discovered that PCNSL typically shows a more uniform APTW hypersignal compared to HGG. Their research identified APTW max-min (the difference between the maximum and minimum APT-weighted imaging values) as the most effective metric for distinguishing PCNSL from HGG, achieving an area under the ROC curve of 0.963 and an accuracy of 94.1% (Jiang et al., 2016).

Additionally, Ohba et al. (2023) evaluated APT imaging in 14 PCNSL and 27 IDH wild-type glioblastoma cases. Their findings indicated significant differences in the percentile value of APT signals from the 1st to the 20th percentile and the 1–100 APT signal width between the two conditions, with the maximum area under the curve at 0.796 obtained from the 1–100 APT signal width. The sensitivity and specificity values were 64.3 and 88.9%, respectively (Ohba et al., 2023). These studies suggest that APT-weighted imaging is beneficial in distinguishing PCNSL from IDH wild-type glioblastoma, advocating for its use in suspected PCNSL cases to avoid unnecessary aggressive surgical resection, thereby improving the accuracy of differential diagnoses.

### 5.3 Identification of isolated cerebral metastases (SBM)

In neuroimaging, distinguishing solitary brain metastases (SBM) from glioblastoma (GBM) is vital for developing targeted treatment strategies. Advanced imaging techniques are showing promise in improving diagnostic precision. For instance, Yu et al. (2017) analyzed conventional and APTw-weighted imaging in 45 SBM and 43 GBM patients. Their findings revealed that the minimum APTw value (APTwmin) effectively differentiated these tumors, evidenced by a high area under the ROC curve (0.905) and a diagnostic accuracy of 85.2%, underscoring APTw imaging’s utility in distinguishing SBM from GBM (Yu et al., 2017). Additionally, Kamimura et al. (2019) evaluated APT signal intensity (APTSI) in both enhanced areas (EA) and surrounding non-enhanced areas (peritumoral high signal intensity area, PHA). They noted that APTSI in EA accurately differentiated GBM from SBM (Kamimura et al., 2019). Moreover, Chen et al. (2023) team conducted a study with 48 brain tumor patients using conventional MRI, APTw, and ASL scans. By measuring mean APTw values and CBF, they demonstrated the effectiveness of APTw MRI in differentiating SBM from GBM (AUC = 0.864), with the combined use of APTw and CBF values enhancing diagnostic efficiency (Chen et al., 2023). Lastly, Sartoretti et al. (2021) employed radiomics feature extraction from APTw MR Imaging. The study indicated that a multilayer perceptron could distinguish primary glial brain tumors from metastases with an AUC of 0.836 and differentiate WHO grade 4 tumors from grades 2/3 and metastases with an average AUC of 0.797 (Sartoretti et al., 2021). These findings collectively suggest that APTw imaging, alongside emerging techniques, holds substantial promise in differentiating SBM from GBM, offering valuable insights for clinical decision-making and treatment planning.

### 5.4 Identifying progressive multifocal leukoencephalopathy (PML)

Kenneth L. Tyler’s research highlights that Progressive Multifocal Leukoencephalopathy (PML) is a demyelinating brain disease caused by the John Cunningham (JC) virus, typically infecting glial cells. Due to its imaging characteristics, PML can sometimes mimic GBM on MRI scans (Shah et al., 2010). To address this diagnostic challenge, Koike et al. (2022) conducted a retrospective analysis of PML and GBM patients who underwent APT-CEST MRI. They focused on the MTRasym obtained from APT imaging. Their findings indicated that an MTRasym value of 0.015 is a reliable marker to differentiate PML from GBM (Koike et al., 2022) (Table 5).

**Table 5:** Amide proton transfer imaging used in the differentiation of high-grade gliomas from other malignant lesions.

Reference	Method of application	Sample size	AUC	Conclusion
Yu et al. (2017)	APT	88	0.905	APT-weighted MR imaging can be used to distinguish SBMs from GBMs.
Kamimura et al. (2019)	APT	48	NA	APTSI in EAs, but not PHAs, is useful for differentiation between GBMs and SBMs.
Sartoretti et al. (2021)	APT	48	0.836	Radiomics for APTw imaging feasible; distinguishes primary glial tumors from metastases accurately
Koike et al. (2022)	APT	22	0.98	MTRasym values obtained from APT imaging allowed patients with PML to be clearly discriminated from patients with PCNSL or GBM.
Zhang et al. (2022)	APT	50	0.947	APT can be used for the differential diagnosis of meningioma and HGG.
Jiang et al. (2023a,b)	APT	32	0.963	PCNSLs exhibit homogeneous APTW, lower maximum signals; higher MTR signals; APTW heterogeneity best distinguishes them from HGGs.
Chen et al. (2023)	APT, ASL	48	0.797	APTw superior to ASL for SBM vs. GBM differentiation; APTw and ASL combo enhances diagnosis.
Ohba et al. (2023)	APT	27	0.796	APTw imaging distinguishes PCNSL from IDH-wildtype glioblastoma, aiding non-aggressive surgical decisions in relevant cases.

## 6 Conclusion

The CEST is widely applied in diffuse gliomas; however, current research still faces certain limitations. First, in many studies, the selected ROI on images may not completely correspond to the pathological areas (Jiang et al., 2019). Additionally, tumors located near the skull or ventricles can produce artifacts during imaging, leading to measurement errors in APT values. Despite these limitations, CEST imaging should still be able to reflect overall trends in changes. Second, the scanning parameters may not be optimal. High spectral resolution of the Z-spectrum can better differentiate certain components, such as glutamate and creatine/guanidine signals (Su et al., 2022). Additionally, in most studies, only 2D single-slice imaging was performed due to time constraints. Given the intratumoral heterogeneity, this approach may miss important pathological regions. Employing 3D acquisition that covers the entire tumor could potentially resolve this issue (Hou et al., 2023b). As a subtype of CEST imaging, APTw alone, or in combination with other functional imaging techniques, can further enhance the effectiveness of glioma grading, molecular subtyping, and postoperative prognosis prediction for adult diffuse gliomas within the context of the WHO CNS5 classification.

The APT imaging in the diagnosis and treatment of glioma has great potential and is expected to play a key role in the accuracy of future medicine. With the advancement of technology and the accumulation of more clinical data, APT imaging is expected to become an important tool for evaluating gliomas and guiding their treatment.

## Author contributions

H-ZD: Conceptualization, Data curation, Formal analysis, Funding acquisition, Investigation, Methodology, Project administration, Resources, Software, Supervision, Validation, Visualization, Writing – original draft, Writing – review & editing. H-WZ: Writing – review & editing. FL: Data curation, Formal analysis, Resources, Writing – review & editing. YL: Formal analysis, Project administration, Validation, Writing – review & editing. J-HD: Investigation, Software, Writing – review & editing. S-PL: Methodology, Project administration, Writing – review & editing. X-LL: Methodology, Project administration, Resources, Writing – review & editing. BH: Resources, Supervision, Writing – review & editing, W-HL: Formal analysis, Project administration, Writing – review & editing..

## Glossary

APTw: Amide Proton Transfer-weighted
CNN: Convolutional Neural Network
DWI: Diffusion Weighted Imaging
ASL: Arterial Spin Labeling
MRI: Magnetic Resonance Imaging
TuR: Tumor Recurrence
TrE: Treatment Effect
DTI: Diffusion Tensor Imaging
DSC: Dynamic Susceptibility Contrast
DCE: Dynamic Contrast Enhanced
AUC: Area Under the Curve
rCBF: Relative Cerebral Blood Flow
SWI: Susceptibility Weighted Imaging
pcASL: Pseudo-Continuous Arterial Spin Labeling
HGG: High-Grade Glioma
dnsAPT: Dynamic Nuclear Spin Amide Proton Transfer
HSV: hot-spot volumes
PWI: Perfusion Weighted Imaging
APT: Amide Proton Transfer
T: Tesla
MR: Magnetic Resonance
RT: Radiotherapy
ADC: Apparent Diffusion Coefficient
FA: Fractional Anisotropy
nCBV: Normalized Cerebral Blood Volume
